# Reducing uncertainties in global HIV prevalence estimates: the case of Zambia

**DOI:** 10.1186/1471-2458-6-83

**Published:** 2006-04-02

**Authors:** Kumbutso Dzekedzeke, Knut Fylkesnes

**Affiliations:** 1 Central Statistical Office, Lusaka, Zambia; 2 Centre for International Health, University of Bergen, Armauer Hansens Hus, N-5021, Bergen, Norway

## Abstract

**Background:**

The premise for using antenatal care (ANC) clinic data for estimating HIV prevalence in the general population is the finding from community studies in sub-Saharan Africa that total HIV prevalence in pregnant women attending ANC clinics closely approximate levels in the total general population of both women and men aged 15–49 years. In this study, the validity of national level HIV prevalence estimates for the total general population 15–49 years made from ANC clinic and population survey data was assessed.

**Methods:**

In 2001–2002, a national population HIV prevalence survey for women 15–49 years and men 15–59 years was conducted in Zambia. In the same period, a national HIV sentinel surveillance survey among pregnant women attending ANC clinics was carried out.

**Results:**

The ANC HIV prevalence estimates for age-group 15–49 years (rural: 11.5%; 95% CI, 11.2–11.8; urban: 25.4%; 95% CI, 24.8–26.0; adjusted national: 16.9%; 95% CI, 16.6–17.2) were similar to the population survey estimates (rural: 10.8%; 95% CI, 9.6–12.1; urban: 23.2%; 95% CI 20.7–25.6; national: 15.6%; 95% CI, 14.4–16.9). The HIV prevalence urban to rural ratio was 2.2 in ANC and 2.1 in population survey estimates.

**Conclusion:**

The HIV prevalence estimate for the total general population 15–49 years derived from testing both women and men in the population survey was similar to the estimate derived from testing women attending ANC clinics. It shows that national HIV prevalence estimates for adults aged 15–49 years can also be obtained from ANC HIV sentinel surveillance surveys with good coverage when ANC attendance and fertility are high.

## Background

UNAIDS has recommended antenatal care (ANC) clinics sentinel surveillance to measure HIV prevalence trends in generalized epidemics due to problems of acceptability when conducting HIV surveys in the general population [1,2]. The premise for using ANC data for estimating HIV prevalence in the general population is the finding from community studies in sub-Saharan Africa that total HIV prevalence in pregnant women attending ANC clinics closely approximate levels in the total general population of both women and men aged 15–49 years [3-7]. Pregnant women thus seem to represent a random sample of the population with respect to HIV prevalence in a generalised epidemic, and it has been suggested that confidence limits about the size of the epidemic become narrower as the epidemic progresses[8,9].

Country estimates of HIV prevalence derived from ANC-based data with the UNAIDS model have faced a barrage of criticisms because estimates from some national population surveys are significantly different from ANC-based estimates [9,10]. Whether these differences are due to errors in surveys or the model has not been demonstrated with countries data.

In Zambia, parallel population and ANC-based HIV prevalence estimates provided from selected surveillance sites have revealed a close match between them [5-7]. These are findings from settings where fertility rates were high and more than 90% of women had made at least one visit to an ANC clinic when pregnant [10]. High validity of ANC-based HIV prevalence estimates at site levels, however, is no guarantee for respective high validity of estimates at national levels. Validity at that level will to a great extent be influenced by the selection of surveillance sites. In 2001–2002, a national HIV prevalence population survey was carried out in Zambia as part of the Zambia Demographic and Health Survey (ZDHS). A national ANC HIV sentinel surveillance survey was also carried out in the same period. This offers an opportunity to compare and validate the representativeness of national ANC-based and population surveys HIV prevalence estimates.

## Methods

### Data sources

#### ANC sentinel surveillance

Repeated cross-sectional surveys in which women on their first ANC clinic visit and accepting routine syphilis testing are anonymously tested for HIV, have been carried out in 1994, 1998 and 2001–2002 in selected ANC sites. They targeted to test about 500 women per site within four months. Similar test protocols have been used in these surveys [7,11,12]. The 2001–2002 survey was from September 2001 to April 2002. Some sites took more than four months and were unable to test the target number. Some urban sites tested over 500 women.

#### The national population HIV survey

The survey was carried out from November 2001 to May 2002 as part of the ZDHS. The sample universe was women 15–49 years living in households [13]. The sample frame was the geographical distribution of household clusters from the 2000 Census of Population and Housing. At least 85 households were in a cluster. With a minimum cluster take of 25 completed interviews of women, 320 clusters were allocated proportional to the population size of provinces within urban and rural areas. One hundred urban and 220 rural clusters were selected at the first stage. At the second stage, households were selected after field listing to update the household information in the selected clusters. De-facto household members of women 15–49 years in 8200 selected households and men 15–59 years in a third of these households were eligible to be interviewed. Women and men in households from which men were selected were eligible for an HIV test.

If a respondent consented to HIV testing, a laboratory technician prepared a dried blood spot (DBS) sample on a filter paper card from a venous blood draw at the household. A three stage test protocol was also used. After eluting the DBS samples, they were first tested using Wellcozyme HIV 1&2 GACELISA. All the positive samples were re-tested using BIONOR HIV 1&2. Discordant cases were tested with Western Blot. A total of 3961 samples were collected, of which 710 tested positive on GACELISA. After testing with BIONOR, 570 cases remained positive. With Western Blot, all 140 discordant samples were confirmed to be negative with the exception of one. In quality control, 10% of the total samples found negative with GACELISA test were re-tested with BIONOR. Two cases were positive on BIONOR as well as on Western Blot. In other quality control, both plasma and DBS samples for 505 respondents were tested. Plasma and DBS samples found positive were 118 and 121 respectively. With Western Blot, the 3 discordant results were negative for the plasma but positive for the DBS samples.

### Ethical aspects

Protocols for both surveys were approved by the Ethical Review Committee of the University of Zambia. The Institutional Review Board of ORC Macro in the USA also approved the ZDHS protocol. Informed consent was sought from all willing participants. Participants were included in the study after obtaining an informed consent. In the ZDHS, additional consent was obtained from the parent or guardian if the respondent was aged 15–17 years.

### Data analysis

Analysis was restricted to women and men aged 15–49 years in the ZDHS. It was also restricted to women in this age group for the ANC sites. Data was analyzed with the Statistical Package for Social Sciences (SPSS for Windows; SPSS, Chicago, Illinois, USA) and the Microsoft Office Excel Spreadsheet (Microsoft Incorporation; Washington USA). We compared HIV prevalence estimates for the general population from the ANC survey and ZDHS by age, sex, province and residence. The 95% confidence intervals (CI) for the ZDHS took into account the design effect of cluster sampling. Cluster effect was not taken into account for pooled ANC sites data because they were selected purposively.

Weights were applied to the ZDHS data in order to adjust for differential non-response by sex and province. Other variables could not be adjusted for because information was not collected in the survey. A sensitivity analysis was carried out in order to assess the potential bias from non-response. The critical level of difference in prevalence levels between respondents and non-respondents which would significantly change the HIV prevalence estimates derived from respondents if non-respondents were also tested was determined.

Total ANC-based HIV prevalence estimates were adjusted for the distribution of the total population by respective age groups between urban and rural areas because 61.8% of the ANC population were from urban areas. However, total population aged 15–49 years that lived in urban areas was 38.6%. The 2000 Zambia census of population and housing was used as the standard population. Most surveys carried out in Zambia in the period these surveys were carried out used it as the sample frame.

## Results

Differential non-response by sex and province had no effect on the total HIV prevalence estimates by age, sex and residence. Changes in the HIV prevalence estimates after weighting were insignificant. Table 1 shows critical differences in HIV prevalence between respondents and non-respondents that would significantly change the HIV prevalence estimates for the general population made from respondents if non-respondents were also tested. Non-response would significantly bias the HIV prevalence estimate for Zambia if HIV prevalence among non-respondents was different by at least 41% from the level among respondents. If this was the case, the HIV prevalence for Zambia would be 17.3%. The critical prevalence difference between respondents and non-respondents increases as the sample size reduces. It ranges from 41% for Zambia with a sample of 3804 respondents, to 289% in North-Western province with a sample of 223 respondents. Sample size, response rate and the prevalence level determine this critical difference

Table 2 shows that non-response in the ZDHS was systematic by age, sex and area of residence. Overall, a weighted total of 2148 women 15–49 years (76.4% of eligible women) and 1757 men 15–49 years (72.4% of eligible men) voluntarily gave blood for HIV testing. The percentage that refused the test was similar between women and men. The proportion of women and men tested was lower in urban than in rural areas. The difference in the proportions was bigger for men than women. The percent tested among men tended to drop with an increase in age in urban areas and to increase with an increase in age in rural areas. Due to a pattern in the response rates by age, the HIV prevalence estimates were standardized for the 2000 Census population age distribution. The urban to rural and women to men ratios for the standardized estimates were only slightly different from those obtained before standardization. The women to men ratio of HIV prevalence increased from 1.4 to 1.7 in urban areas, to 1.5 in rural areas and to 1.5 for the total population. HIV prevalence among women and men in urban areas was respectively about twice the level of that in rural areas. This pattern persists in all age groups for women but only in the 25–29 and 30–39 years age groups for men. Peak prevalence was in the 30–39 years age group for women in urban and rural areas. It was also in this age group for men in urban areas but in the older age group of 40–49 years in rural areas.

**Figure 1:**
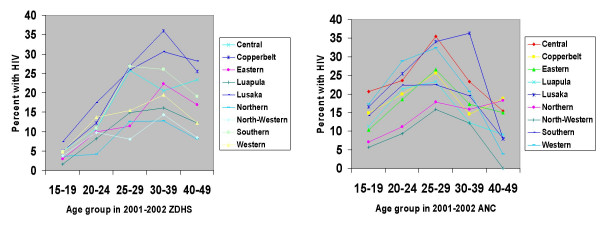
2001–2002 ANC and ZDHS HIV prevalence estimates by age group and province.

Table 3 shows that the age patterns for ZDHS and ANC-based prevalence estimates were different although their total estimates were not significantly different. Their total urban to rural prevalence ratios at 2.2 for ZDHS and 2.1 for ANC-based estimates were close. ANC-based estimates were higher than ZDHS estimates in the age groups 15–19, 20–24 and 25–29. Peak HIV prevalence was in the age group 30–39 years in the ZDHS and earlier in the 25–29 year age group for ANC-based estimates. Figure 1 shows a similar pattern among the provinces. Peak prevalence was mainly in the 25–29 years age group in ANC-based estimates and 30–39 years age group in ZDHS estimates.

Table 4 shows that the ZDHS and ANC-based HIV prevalence estimates for urban and rural areas of provinces were also close. They were only significantly different for rural areas in Central and Southern provinces. In rural Central province, the ANC-based estimate was significantly higher. In rural Southern province, the ZDHS estimate was more than twice as high as the ANC-based estimate. The cut-off point for domain samples which would yield plausible estimates of HIV prevalence in the general population is about 627. This yielded an HIV prevalence estimate with a 20% variation within 95% CI in urban Copperbelt province. Smaller domain samples did not have the power to yield estimates with a better precision. ANC-based estimates derived from larger samples and not affected by non-response because testing for HIV was anonymous, had a variation of less than 10% within the 95% CI for all domains. The variation of the ZDHS estimates within the 95% CI ranges from 8.3% for total Zambia to 83.2% for rural Copperbelt province while the variation of all the ANC-based estimates was within 10% of the 95% CI. Mean ANC-based HIV prevalence estimates for the general population tended to be higher than those from the ZDHS.

## Discussion

The HIV prevalence for the total population 15–49 years in 2001–2002 was 15.6% from the ZDHS and 16.9% from the ANC-based data. ZDHS estimates indicated an HIV prevalence of 13.0% among men and 17.8% among women. The prevalence ratio of women to men was 1.4 in both urban and rural areas. ZDHS estimates for urban and rural areas were 23.2% and 10.8% respectively. The respective ANC-based estimates were 25.4% and 11.5%. The urban to rural prevalence ratios were 2.1 for ZDHS and 2.2 for ANC-based estimates. ANC-based estimates for the general population of provinces were also not significantly different except for total Central province, rural areas of Central province and rural areas of Northern province.

We conclude that there was no significant difference in the total, urban and rural HIV prevalence estimates derived from the two data sources. The similarity was not by chance as shown by the similarity of ANC-based and ZDHS estimates for all provinces but one, total urban, total rural, all urban areas and seven rural areas of the provinces. It showed that both ANC-based and population survey estimates can be similar even at national level if there are no coverage distortions in the national surveys in countries with generalized epidemics. ANC sites estimates also gave plausible indications of the level of HIV prevalence in the general population of the provinces without coverage distortions. The difference in estimates for rural areas of Central province and Southern province could be due to the disproportionate percentage of their clients who lived in urban areas. In Central province, 19.7% of ANC clients lived in urban areas and only 0.6% in the rural site of Southern province [14].

Provincial HIV prevalence estimates from the ZDHS would be more reliable than ANC-based estimates because they are less affected by coverage bias. Unbiased coverage in the ZDHS is shown in many ways. Age standardized estimates were only slightly different from the non standardized estimates. There was a similarity between weighted and un-weighted HIV prevalence estimates. Significant differences would indicate bias in the coverage of some groups. However, weighting can not adjust for any differences in the HIV prevalence of the respondents and non-respondents. Such differences can only be assessed through an independent survey of those persons who were not covered in the ZDHS. HIV prevalence in Zambia increases by level of education. However, differences in response rates by level of education in the ZDHS were small and they were not in any direction[13]. Other studies have shown that the association between level of education and infection changed between 1995 and 2003 in some communities in Zambia, from being clearly positive to being negative in age-groups younger than 30 years[15]. A population survey in catchment areas of some ANC sites also showed that neither mobility nor migration was associated with HIV infection [7]. Therefore, HIV prevalence of absent respondents was less likely to be different from that of respondents. Further, the margin of difference in prevalence levels between respondents and non-respondents required to significantly change the prevalence estimates if non-respondents were also tested is too big for non-respondents without an elevated risk of being infected. Other surveys have shown that HIV prevalence levels of non-respondents are not significantly different from those of respondents unless the exposure variables of non-respondents or HIV test results from the sample frame indicate an elevated risk of contracting HIV [16,17].

ANC-based and ZDHS estimates of HIV prevalence for the total general population show that ANC-based estimates were higher in the age groups 15–19, 20–24 and 25–29 years by 58.4%, 35.4% and 10.2% respectively. They were lower by 37.0% and 79.3% in age groups 30–39 and 40–49 years respectively. This pattern does not occur by chance. It was also seen in the provinces. Misrepresentation of prevalence by age group in the general population by ANC-based estimates cancelled itself out almost completely. Total ANC-based and ZDHS estimates were similar at the national level just as has been observed in some communities.

WHO/UNAIDS using an epidemiological model with ANC-based data for inputs estimated total HIV prevalence for adults 15–49 years in Zambia to be 19.1% in 1997 [18], 20.0% in 1999 [19] and 21.5% in 2001[20]. Compared to our ANC-based estimate, they over-estimated the Zambian epidemic by 27% (16.9% versus 21.5%) in 2001. However, in the 2003 update, it was changed from 21.5% to 16.5% [21].

The dynamic interactions of HIV and its host population which affects fertility and mortality and in turn HIV prevalence as well has been reported for Eastern Africa [22]. ANC-based estimates are initially close to population based estimates when the female to male HIV prevalence estimates are less than 1.4. Afterwards prevalence from ANC-based estimates could become biased downwards with the increasing average duration of the infected population and the increase in fertility impairment with duration even when there has been no change in prevalence in the general population[22].

Marked HIV declines in young men and women in the general population were understated in the parallel ANC-based trends in some communities in Zambia over a shorter period from 1995 to 2003 [15]. Explanations for this trend were related to changes in fertility behaviours among young women, i.e. postponement of child bearing, and differential HIV declines by level of education. Because of such changes in the demographic state induced by the HIV epidemic, methods and models for measuring HIV transmission trends might need to be changed [22,23]. Household surveys, despite their high cost could be needed as alternative systems for measuring HIV prevalence in the general population. High non-response might appear a threat in this regard, however, and the well functioning ANC-based systems should continue until alternative systems are in place.

## Conclusion

It is apparent that ANC-based surveillance can provide total national HIV prevalence estimates for adults in the general population aged 15–49 years that match those from population surveys for a country with a generalized HIV epidemic, high ANC coverage, fertility levels and good coverage of ANC-based HIV surveillance surveys. Differences observed between national ANC-based and population survey HIV prevalence estimates for some countries with generalized epidemics could be due to coverage distortions in the surveys. Over the course of the epidemic, population surveys might be the best source of data for monitoring prevalence in the general population.

## Competing interests

The author(s) declare that they have no competing interests.

## Authors' contributions

KD was the principal investigator for the 2001–2002 ZDHS and made substantive contributions to the design of this survey and directly participated in the development of the data collection strategy, costing of the survey, training of all levels of survey staff, supervising the fieldwork and data analysis. The data from the 2001–2002 ANC-based HIV Surveillance system were made available from the Central Board of Health and for the purpose of this paper analysed by KD. KF contributed substantially in designing and developing the national ANC-based HIV surveillance system and the establishment of parallel population-based surveys in selected sites, and contributed all through the preparation of the paper.

## Pre-publication history

The pre-publication history for this paper can be accessed here:



## Figures and Tables

**Table 1:** Difference in HIV prevalence between respondents and non-respondents that could change 2001–2002 ZDHS estimates

Province	Total women and men eligible for an HIV Test	Percentage of women and men with test results	HIV prevalence estimated from tested women and men	Minimum percent difference in prevalence between respondents and non- respondents that significantly changes total prevalence estimated from respondents	Minimum significant change in estimated prevalence if it was different by the minimum percentage between respondents and non-respondents	HIV prevalence if prevalence was higher by the stated minimum percentage among non-respondent women and men
Central	402	75.9	15.4	157	5.8 [0.1–11.5]	21.2
Copperbelt	1052	73.7	19.9	74	3.9 [0.1–7.7]	23.8
Eastern	714	65.8	13.8	91	4.3 [0.1–8.5]	18.1
Luapula	359	83.3	11.3	283	5.4 [0.1–10.7]	16.7
Lusaka	778	71.9	22.0	76	4.7 [0.1–9.3]	26.7
Northern	698	74.1	8.3	159	3.4 [0.1–6.8]	11.7
North-Western	223	74.4	9.0	289	6.6 [0.1–13.0]	15.6
Southern	489	83.4	17.6	186	5.4 [0.1–10.6]	23.0
Western	425	71.8	13.1	146	5.4 [0.1–10.7]	18.5
						
Zambia	5140	74.0	15.6	41	1.7 [0.1–3.2]	17.3

*Use of weights causes slight differences with figures in Table 3

**Table 2:** Weighted response rates and HIV prevalence among women and men 15–49 years, 2001–2002 ZDHS.

Residence	Number eligible for HIV test		Percent tested		Percent that refused		Percent absent		Percent HIV positive [95% confidence intervals]		Women to men HIV prevalence ratio
						
	Women	Men	Women	Men	Women	Men	Women	Men	Women†	Men†	ratio
Urban											
15–19	210	206	80.8	74.8	12.3	13.6	6.9	11.7	8.8 [5.5–14.3]	2.2 [0.6–9.2]	4.0
20–24	197	173	69.9	73.4	22.7	11.0	7.4	15.6	22.8 [16.6–30.2]	4.9 [2.0–11.1]	4.7
25–29	175	159	76.4	66.7	14.8	16.4	8.7	17.0	38.3 [30.2–47.2]	24.2 [16.1–34.3]	1.6
30–39	178	289	75.7	66.4	14.0	15.6	10.2	18.0	41.7 [33.6–50.2]	34.0 [26.7–42.4]	1.2
40–49	90	137	73.2	59.1	18.7	21.9	8.1	19.0	25.3 [16.7–36.3]	28.4[17.8–1.59	0.9
											
Total	850	964	75.3	68.5	16.5	15.4	8.2	16.2	26.2{27.4} [23.0–29.8]	19.2{16.6} [15.9–23.1]	1.4
											
Rural											
15–19	433	339	70.7	70.2	20.3	18.6	9.0	11.2	4.6 [2.8–7.9]	1.7 [0.7–3.4]	2.7
20–24	361	218	80.1	72.5	12.5	13.8	7.5	13.8	11.8 [8.5–16.5]	4.2 [1.8–8.7]	2.8
25–29	287	247	76.7	77.7	18.1	13.4	5.2	8.9	15.3 [11.2–20.8]	9.6 [6.0–14.7]	1.6
30–39	388	382	82.5	77.0	13.4	11.5	4.1	11.5	18.4 [14.4–23.1]	12.5[9.0–16.99	1.5
40–49	212	278	77.4	77.0	17.0	12.2	5.7	10.8	11.3 [7.4–16.4]	16.7 [11.7–23.6]	0.7
Total	1681	1464	77.3	74.9	16.2	13.9	6.5	11.2	12.3{12.1} [10.6–14.3]	9.0{8.0} [7.3–10.8]	1.4
											
Total											
											
15–19	693	545	74.5	72.1	17.3	16.7	8.2	11.2	6.6 [4.6–9.3]	1.9 [0.9–4.0]	3.5
20–24	644	391	75.5	72.9	16.9	12.5	7.6	14.6	16.3 [12.9–20.3]	4.5 [2.5–7.8]	3.6
25–29	517	406	76.4	73.4	16.8	14.5	6.8	12.1	25.1 [20.7–30.1]	15.1 [11.1–20.0]	1.7
30–39	623	671	79.9	72.4	13.6	13.3	6.4	14.3	26.4 [22.5–30.9]	21.4 [17.5–25.8]	1.2
40–49	334	414	76.0	71.3	17.4	15.2	6.6	13.5	15.7 [11.7–20.6]	20.4 [15.4–26.6]	0.8
											
Total	2811	2427	76.4	72.4	16.3	14.5	7.2	13.1	17.8{17.5} [16.1–19.6]	13.0{11.9} [11.2–14.8]	1.4

† Figures in {} are standardized for the 2000 census population age

**Table 3:** General population HIV prevalence estimates by age from the 2001–2002 ZDHS and ANC SS

Survey/Province	Number with HIV test results*		Percentage with HIV [95% confidence intervals]		
		
	Urban	Rural	Total	Urban	Rural
2001–2002 ZDHS Women and men*					
					
15–19	349	514	4.6 [3.3–6.3]	6.6 [4.1–10.2]	3.3 [2.1–5.1]
20–24	321	431	11.4 [9.2–14.1]	15.0 [11.0–19.8]	8.8 [6.5–12.0]
25–29	274	407	20.4 [17.3–23.9]	32.1 [26.1–38.7]	12.5 [9.6–16.1]
30–39	369	601	23.9 [21.1–27.0]	37.7 [32.0–43.5]	15.5 [12.8–18.7]
40–49	171	369	18.0 [14.6–21.7]	26.9 [19.7–35.0]	13.8 [10.6–17.9]
					
Total	1484	2322	15.6{15.1} [14.4–16.9]	23.2{22.8} [20.7–25.6]	10.8{10.3} [9.6–12.1]
					
2001–2002 ANC women					
15–19	1767	1157	11.1(13.2) [10.7–1.5]	16.9 [16.1–17.7]	7.6 [7.2–8.0]
20–24	2606	1430	17.6(20.7) [17.1–8.1]	25.0 [24.1–25.9]	12.7 [12.1–13.3]
25–29	1686	949	22.7(26.9) [21.9–3.5]	33.3 [31.7–34.9]	15.6 [14.6–16.6]
30–39	1251	934	17.4(20.9) [16.7–18.1]	28.7 [27.1–30.3]	10.4 [9.8–11.0]
40–49	95	114	10.6(11.0) [9.2–12.0]	13.7 [11.0–16.4]	8.8 [7.3–10.3]
					
Total	7405	4584	16.9(20.1) [16.6–17.2]	25.4 [24.8–26.0]	11.5 [11.2–11.8]

*Use of weights caused slight differences with figures in Table 1 and Table 4.

Totals in () are unadjusted for the urban-rural distribution of the total population of women and men aged 15–49 years from the 2000 Census of Population and Housing.

Figures in {} are standardized for the 2000 census population five year age group distribution for adults aged 15–49 years old

**Table 4:** Adult general population HIV prevalence estimates by province from 2001–2002 ZDHS and ANC SS

Survey/Province	Number with HIV test results*		Percentage with HIV [95% confidence intervals]		
	Urban	Rural	Total	Urban	Rural
2001–2002 ZDHS Women and men*					
					
Central	71	234	15.4 [12.6–18.6]	25.4 [18.9–34.0]	12.4 [9.4–15.7]
Copperbelt	627	148	19.9 [16.6–23.8]	22.3 [18.5–26.8]	9.5 [5.0–17.4]
Eastern	59	411	13.8 [10.6–17.6]	20.3 [11.6–34.5]	12.7 [9.5–16.8]
Luapula	42	257	11.3 [8.2–15.2]	21.4 [12.3–36.4]	9.3 [6.5–13.5]
Lusaka	447	112	22.0 [18.3–26.2]	23.3 [19.1–28.1]	17.0 [10.3–26.6]
Northern	86	431	8.3 [6.3–10.8]	25.6 [17.7–34.8]	4.9 [3.3–7.2]
North-Western	24	142	9.0 [6.8–12.3]	16.7 [9.9–28.8]	7.7 [5.4–11.0]
Southern	87	321	17.6 [14.1–21.8]	21.8 [14.1–31.8]	16.5 [12.8–21.2]
Western	38	267	13.1 [9.6–17.6]	34.2 [19.5–50.8]	10.1 [7.0–14.7]
Zambia	1481	2323	15.6{15.1} [14.4–16.9]	23.2{22.8} [20.7–25.6]	10.8{10.3} [9.6–12.1]
					
2001–2002 ANC women					
					
Central	497	525	23.8(25.2) [22.4–25.2]	28.8 [26.3–31.3]	21.9 [20.1–23.7]
Copperbelt	994	393	20.1(19.0) [19.1–21.1]	22.7 [21.3–24.1]	9.7 [8.8–10.6]
Eastern	450	523	11.9(18.0) [11.2–12.6]	27.1 [24.6–29.6]	10.1 [9.3–10.9]
Luapula	495	802	16.8(17.2) [15.9–17.7]	22.2 [20.3–24.1]	14.1 [13.2–15.0]
Lusaka	2942	-	27.2(27.2) [26.2–28.2]	27.2 [26.2–28.2]	-
Northern	519	520	8.5(12.5) [8.0–9.0]	18.3 [16.8–19.8]	6.7 [6.2–7.2]
North-Western	495	878	8.8(10.0) [8.4–9.2]	13.5 [12.4–14.6]	8.0 [7.5–8.5]
Southern	519	520	13.6(19.6) [12.8–14.4]	31.6 [28.9–34.3]	7.7 [7.1–8.3]
Western	494	423	16.8(23.8) [15.7–17.9]	31.8 [29.0–34.6]	14.4 [13.1–15.7]
					
Zambia	7405	4584	16.9(20.1) [16.6–17.2]	25.4 [24.8–26.0]	11.5 [11.2–11.8]

*Use of weights accounts for the slight differences with figures in Table 3.

Totals in () are unadjusted for the urban-rural distribution of the total population of women and men aged 15–49 years from the 2000 Census of Population and Housing.

Figures in {} are standardized for the 2000 census population five year age group distribution for adults aged 15–49 years old
